# Characterization and Evaluation of Silver Concentrations in Hydroxyapatite Powders

**DOI:** 10.3390/jfb14090467

**Published:** 2023-09-11

**Authors:** Néstor Méndez-Lozano, Miguel Apatiga-Castro, Alvaro de Jesús Ruíz-Baltazar, Miguel de la Luz-Asunción, Eduardo E. Pérez-Ramírez

**Affiliations:** 1 Campus Querétaro, Universidad del Valle de México, Blvd. Juriquilla No. 1000 A Del., Santa Rosa Jáuregui 76230, Querétaro, Mexico; miguel_delaluz@my.uvm.edu.mx (M.d.l.L.-A.); eduardo_perezr@my.uvm.edu.mx (E.E.P.-R.); 2 CONAHCYT—Centro de Física Aplicada y Tecnología Avanzada, Universidad Nacional Autónoma de México, Boulevard Juriquilla 3001, Santiago de Querétaro 76230, Querétaro, Mexico; apatiga@unam.mx (M.A.-C.); aruizbaltazar@fata.unam.mx (A.d.J.R.-B.)

**Keywords:** antibacterial evaluation, biomedical applications, hydroxyapatite powders, crystalline structure

## Abstract

The goal of this study is to evaluate the influence of the concentration of silver on the structural and antimicrobial in vitro properties of silver-doped hydroxyapatite powders obtained using the precipitation method. Different concentrations of silver were evaluated to assess the antimicrobial properties. X-ray diffraction (XRD), Fourier transform infrared spectroscopy (FTIR), Raman spectroscopy, scanning electron microscopy (SEM), and dispersive energy spectroscopy (EDS) were used to characterize the powders. XRD and FTIR showed that the hydroxyapatite structure is not affected by the incorporation of silver; on the other hand, EDS showed the presence of silver in the powders. Antibacterial studies showed the efficiency of hydroxyapatite powders in inhibiting bacterial growth as silver concentration increases. According to the results, silver-doped hydroxyapatite powders are suggested for use in the prevention and treatment of infections in bone and dental tissues.

## 1. Introduction

The use and research of biomaterials comprise an area of great interest today because this area involves materials science and biological science to achieve an interaction between the body and new materials. As has been reported in several works, hydroxyapatite has good biocompatibility and osteoconductivity, being widely used in dental implants and for bone tissue regeneration [1]. On the other hand, infections and risks of toxicity are some of the problems with the use of medical implants, or the body rejects the implant. Today, in most medical implants, biocompatible coatings are used to avoid problems such as infections, and hydroxyapatite is one of the most used materials for these purposes [2,3,4,5].

Bones and teeth are made up of an organic phase, mainly type I collagen, and another inorganic phase, mostly hydroxyapatite. For this reason, hydroxyapatite is widely used for bone tissue regeneration because of its excellent properties, such as biocompatibility. The chemical composition of hydroxyapatite is like that present in natural bone, also including some ionic substitutions [6,7].

Several investigations have shown that the incorporation of ions, such as Cu, Ag, or Zn, within the hydroxyapatite structure can modify its biological, physical, and chemical properties. Mainly, the incorporation of metallic silver ions in the hydroxyapatite structure has shown an improvement in the physicochemical properties of the material [8,9].

Several investigations report the use of silver ions or metallic silver in different medical applications, such as the treatment of burns, dental materials, textile fabrics, wastewater treatment, sunscreens, etc. [10,11]. The main applications of materials with antimicrobial effects are found in medicine. Some researchers have shown that hydroxyapatite is the ideal material for doping with silver ions. Hydroxyapatite has a hexagonal structure formed by type 1 (trigonal tripoint prisms) and type 2 (pentagonal pyramids) calcium, so silver ions can replace calcium sites. The substitution in the structure suggests an improvement in the antibacterial and biocompatibility properties [12,13,14]. On the other hand, other researchers reported HApTiAg and HApTiAg coatings with high efficiency in the destruction of *Escherichia coli* using different light exposure parameters. Recent studies have shown a high antibacterial activity of hydroxyapatite materials coated with silver and titanium obtained using a chemical method [15]. Even though hydroxyapatite powders have good biocompatibility, one of the most important problems today when using implants for bone or dental tissue regeneration is the risk of infection.

Different investigations to develop new materials with high biocompatibility and antimicrobial properties have been carried out because of the high mortality rates from infections. Silver has been a widely used material since antiquity because of its antimicrobial properties since it attacks a wide range of microorganisms [16]. According to some authors, silver is toxic at high concentrations, but at low concentrations, it is an excellent antimicrobial agent. Silver can bind to a wide variety of cellular components, its interaction with the components of the cell membrane being more important [17]. As mentioned above, hydroxyapatite can substitute type 2 calcium ions with other metal ions, such as silver Ag+, without the substitution changing the structure and properties of the hydroxyapatite. Finally, some researchers have demonstrated that silver-doped hydroxyapatite nanoparticles obtained with the precipitation method at 100 °C have high effectiveness as antimicrobial agents against gram-positive and gram-negative bacteria [18]. Different investigations provide information on the physicochemical and biological properties of hydroxyapatite in relation to ionic substitutions in the structure [19,20,21]. Hydroxyapatite implants alone have some disadvantages since proteins and some organic substances are easily adsorbed, and this can promote the growth of bacteria on the implant surface, which can lead to infections and less penetration of antibiotics [22]. Microorganisms can form biofilms when found on the surface of implants; this condition can make them more resistant to antimicrobial agents [23].

On the other hand, it is well known that implant materials inside the human body interfere with the body’s defense mechanisms. Therefore, they influence the doses of antibiotics that are needed to protect against infections. In addition, many antibiotics are rapidly eliminated by body fluids and, therefore, cannot prevent infection after long-term surgery. In recent years, some research has reported the use of doped hydroxyapatite for possible bone regeneration applications. Studies show the use of the precipitation method to obtain silver-doped hydroxyapatite paste. However, the samples present the presence of silver phosphate, indicating that there is no substitution of ions within the structure. To eliminate the presence of silver phosphorus, a temperature of 1000 °C is necessary [24,25,26]. Currently, silver-doped hydroxyapatite powders have different practical uses, such as dental or orthopedic implants.

Table 1 shows a comparison of the advantages and disadvantages of different methods to obtain silver-doped hydroxyapatite. Our work uses the wet precipitation method, which has the advantage of being low-cost and reproducible, as well as friendly to the environment in the synthesis process. Its disadvantages are the control of morphology and the possible mixture of phases, however. This work seeks to better control these parameters.

The goal of our study is to obtain a reproducible and low-cost route for the synthesis of silver-doped hydroxyapatite powders without the need to use high temperatures in the calcination phase, evaluating the effect of silver concentration on the structure, morphology, and antibacterial properties of hydroxyapatite powders for their possible application in dental implants. On the other hand, it is necessary to consider the moderate use of silver to avoid cytotoxicity problems.

## 2. Materials and Methods

### 2.1. Synthesis of Silver-Doped Hydroxyapatite Powders (HApAg)

Materials used include calcium nitrate [Ca(NO_3_)_2_], ammonium phosphate [(NH_4_)H_2_PO_4_], and silver nitrate [AgNO_3_]. All reagents used are analytical grade, and deionized water was used throughout the synthesis process.

The synthesis of silver-doped hydroxyapatite powders (HApAg) was carried out using the sol-gel method. The precursors used were calcium nitrate [Ca(NO_3_)_2_], ammonium phosphate [NH_4_H_2_PO_4_], and silver nitrate [AgNO_3_]. A calcium nitrate solution with a 1 M concentration was prepared, and silver nitrate was added in different concentrations. In addition, an ammonium phosphate solution of 0.48 M was prepared under constant stirring. The calcium nitrate solution with silver nitrate was dripped at 0.3 mL/min onto the ammonium phosphate solution; the pH was adjusted to 11 using a solution of aqueous ammonia. The separating funnel containing the calcium nitrate and silver nitrate solution was covered with aluminum foil, and the reaction was carried out in a dark vessel because of the photosensitive properties of silver nitrate. After the dripping was complete, the precipitate was continued under constant stirring for a period of 2 h at 500 rpm. Finally, it was left to rest for three days at room temperature; then, it was filtered using filter paper and washed with deionized water to dry at 100 °C for 2 h.

**Figure 1:**
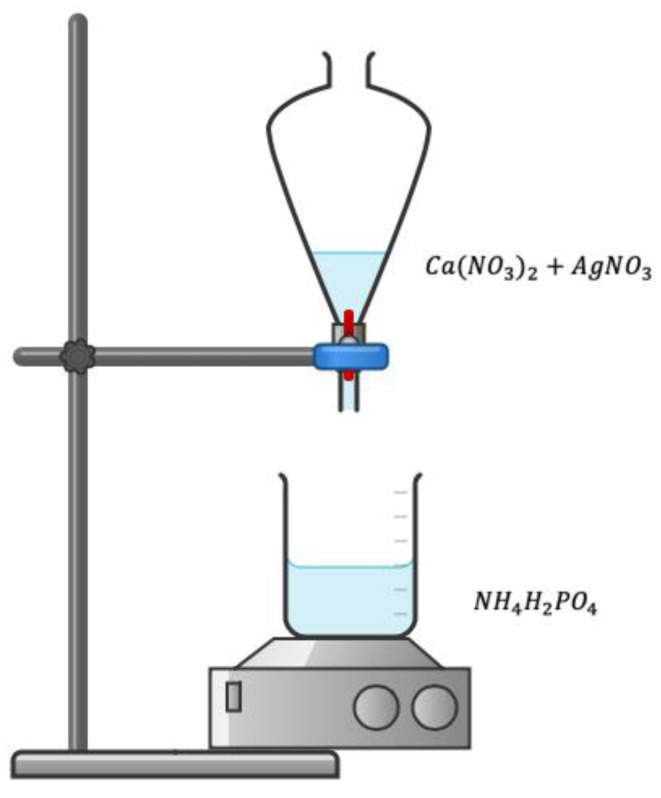
Diagram of the synthesis process of HApAg.

The powders obtained were homogeneous and white in color. For this work, four samples with different concentrations of silver were prepared. The samples were selected by sweeping different concentrations of silver and avoiding high concentrations. The data from the 4 experiments are shown in Table 2. In Figure 1, a diagram of the synthesis process is shown.

### 2.2. Antibacterial Evaluation

A culture medium (nutrient agar) was established using *Escherichia coli*. Initially, a blank was established where the nutrient agar did not contain the HApAg solution. In the preparation of the media, a 25 mL nutrient agar solution was prepared, and 1.2 mg/mL of HApAg powder solution was added at a temperature of 40 °C. After mixing, they were emptied into Petri dishes and allowed to cool to room temperature. The culture media were exposed to the environment for 10 min before being incubated. Subsequently, they were incubated at a temperature of 37 °C for 48 h. This methodology was carried out for each of the samples.

### 2.3. Functional Groups: Fourier Transform Infrared Spectroscopy (FTIR)

To identify the HAp molecule through its functional groups and to determine its purity, an FTIR spectrometer Bruker model Vector 33 was used. Infrared spectra were recorded in medium infrared (MIR) between 650 and 4000 cm^−1^ with a resolution of 1 cm^−1^. Each sample was mixed with KBr (potassium bromide) in a ratio of 3:1; the powders were ground to form a homogeneous mixture [30].

### 2.4. Vibrational Modes: Raman Spectroscopy

To analyze the characteristic vibrational modes of hydroxyapatite, a Raman spectrometer Bruker model Senterra was used. The operation condition was a voltage of 100 mV, a resolution of 3 to 5 cm^−1^, and a 20× objective. The samples were placed in powder [30].

### 2.5. Morphology and Microstructure: Scanning Electron Microscopy (SEM)

Morphological, topological, and microstructural analyses of all samples were carried out with a JEOL JXA-8530F Scanning Electron Microscope, Queretaro, Mexico. The analysis was performed using 20 kV electron acceleration voltage, and secondary electrons formed the images. All the samples were placed on a stainless-steel plate with a separation of 5 mm, pasted with silver paint, and covered with a thin gold film by sputtering to avoid electrostatic charge accumulation [30].

### 2.6. Elemental Composition: Dispersive Energy Spectroscopy (EDS)

To obtain the elemental composition of HAp-Ag powders, an electronic microprobe for microanalysis (EPMA) JXA-8530F was used. The operation conditions were a voltage of 10 kV and a current of 0.10 nA. A small tablet was made with each sample, and then it was coated with a thin graphite film [30].

### 2.7. Phase Composition: X-ray Diffraction (XRD)

X-ray powder diffraction was used to identify the crystalline phases contained in all samples. Wide-angle X-ray experiments were carried out with a Rigaku Mini Flex diffractometer using the Cu $k_{\alpha }$ radiation (λ=1.5406Å), an accelerating voltage of 40 kV, and a current of 30 mA. Diffractograms were recorded with a Solid-State D/teX-ULTRA Detector from 5 to 80° on a 2θ scale with a rate of 10° per minute. Spectrum analysis software, MDI Jade V 5.0.37, was used [30].

## 3. Results

### 3.1. Fourier Transform Infrared Spectroscopy (FTIR)

**Figure 2:**
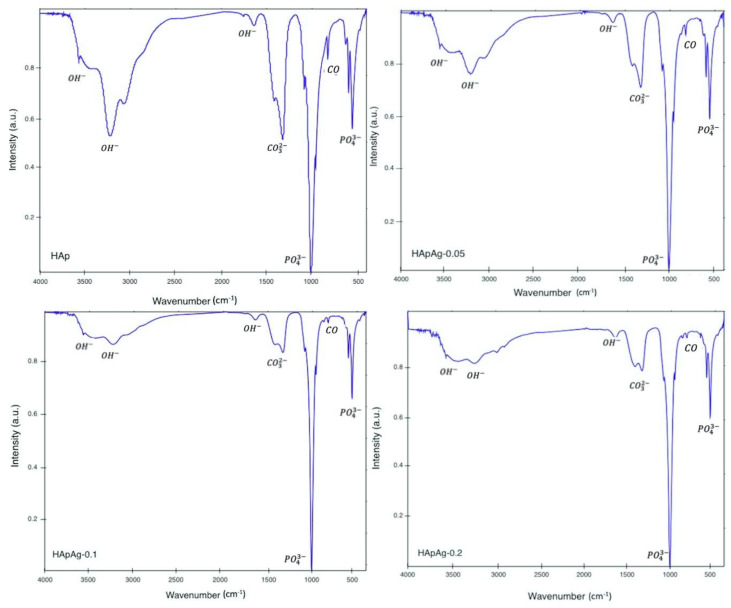
Transmittance infrared spectra of silver-doped hydroxyapatite powders.

FTIR spectroscopy was conducted to evaluate the presence of the functional groups present in the silver-doped hydroxyapatite powders shown in Figure 2. The data revealed the presence of several vibrational modes corresponding to the phosphate and hydroxyl groups. For all samples, a strong vibration peak can be observed, corresponding to the vibrational mode corresponding to ${OH}^{-}$ groups. The bands in the 1600–1700 cm^−1^ and 3200–3500 cm^−1^ regions correspond to the water present in the crystal lattice [31,32,33].

The wide bands are attributed to water absorbed by the samples; however, the bands decrease as the amount of silver Ag increases. The intensity changes in the spectra suggest the incorporation of silver ions ${Ag}^{+}$ in the structure replacing calcium ions ${Ca}^{2+}$. Table 3 shows the absorption bands present in the hydroxyapatite powder samples.

The FTIR spectra present similarly to those reported for hydroxyapatite [34]. In agreement with previous studies, the FTIR studies of silver-doped hydroxyapatite powders show no evidence of a change in crystalline structure [35]. However, previous studies have reported that the silver (I) cation (0.128 nm) is substituted at type 1 calcium sites in the hexagonal lattice of hydroxyapatite. The hexagonal structure of hydroxyapatite is flexible and can incorporate a wide variety of cationic and anionic substitutions [36,37]. In all the spectra, the presence of impurities or secondary products can be observed. At 870 cm^−1^, the presence of the carbonate group is observed. The high crystallinity of the samples is due to the standing/stirring time after adding the additives. In the FTIR spectra of the silver-doped hydroxyapatite samples, a decrease in the intensity of the peaks can be observed with respect to the pure hydroxyapatite spectrum, evidencing that the crystallized hydroxyapatite phase is the only one detected.

### 3.2. Raman Spectroscopy

**Figure 3:**
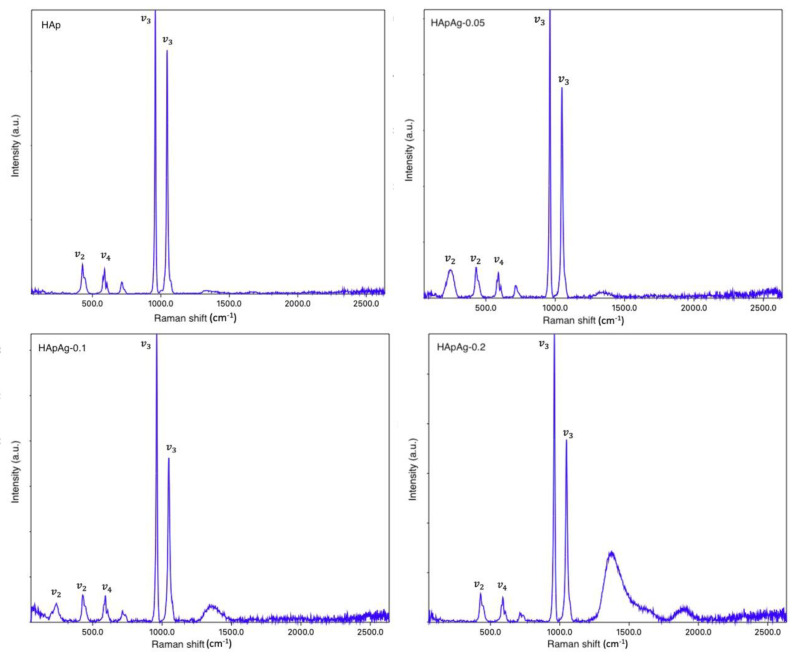
Raman spectra of silver-doped hydroxyapatite powders.

Additional information was obtained from the Raman spectra. The results are shown in Figure 3. The presence of peaks corresponding to the phosphate and hydroxyl groups is observed. The presence of the phosphate group is observed in the vibrational bands around 450 cm−^1^ and 550 cm−^1^, which can be attributed to the ${PO}_{4}^{3-}$ bending mode $v_{2}$. In addition, the maximum peaks that are observed around 1000 cm^−1^ are characteristic of the ${PO}_{4}^{3-}$ stretching mode $v_{3}$. Therefore, the results obtained in the studies of FTIR spectroscopy and Raman spectroscopy agree. In both studies, the purity and crystallinity of the powders can be observed without the influence of silver on the molecular structure of hydroxyapatite. However, as the FTIR studies showed, there is the presence of impurities such as carbonate in the molecular structure.

Raman studies provide information on the hexagonal structure of hydroxyapatite, and there is no presence of peaks related to a secondary phase. However, a decrease in the intensity in the spectra can be observed as the doping concentration increases, indicating a degradation in the crystallization of the samples. According to the literature, as the Ag/Ca partial substitution ratio increases, there appears to be a significant decrease in Raman intensity values due to structural gaps [38,39]. Table 4 shows the Raman shifts observed in the hydroxyapatite powder samples.

### 3.3. Scanning Electron Microscopy SEM

SEM analysis gave information about the morphology of the prepared powders. The morphology of the HApAg-prepared powders with 0 ≤ x ≤ 0.2 was investigated using scanning electron microscopy.

**Figure 4:**
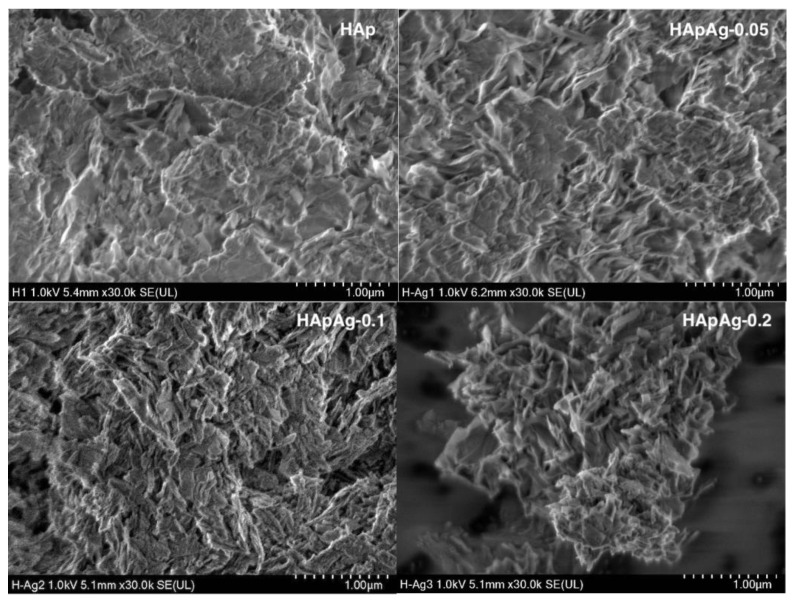
SEM images of silver-doped hydroxyapatite powders at ×30,000. Agglomerated microplates are observed.

**Figure 5:**
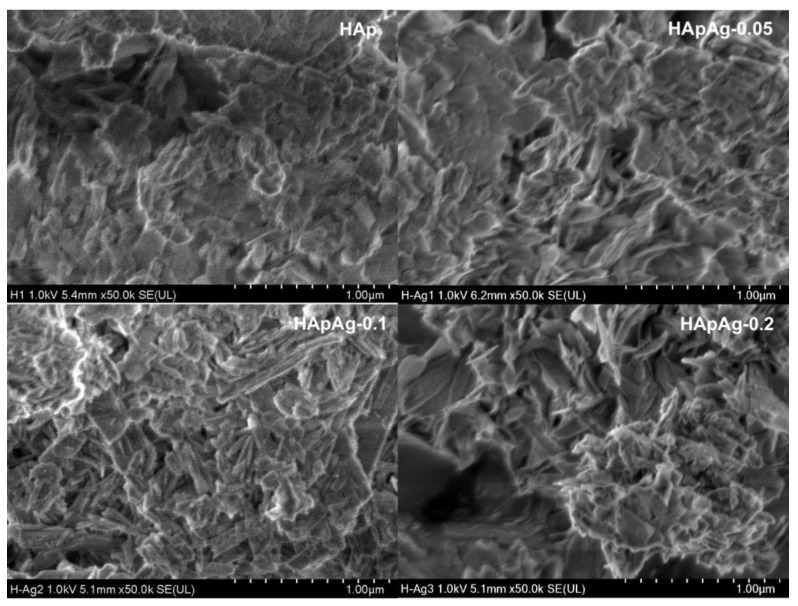
SEM images of silver-doped hydroxyapatite powders at ×50,000.

Figure 4 shows SEM micrographs of HAp, HApAg-0.05, HApAg-0.1, and HApAg-0.2 with a magnification of ×30,000. The results indicate that the addition of silver has no influence on the morphology of the samples. On the other hand, Figure 5 shows SEM micrographs of the same areas with a magnification of ×50,000; the images show plate-like morphology that is agglomerated. This morphology is characteristic of hydroxyapatite obtained using the precipitation method, as has been reported in previous work [40]. All micrographs show a morphology with homogeneous distribution and fine-grained nanostructures. The nanoplate-like morphology is like that present in the structure of human teeth. The width and length of the nanoplates, on average, are on the order of 330 nm and 160 nm, respectively.

The results reveal that the width and length of the nanostructures can vary. However, they suggest that the larger plates are formed by an agglomeration of smaller plates.

### 3.4. Energy Dispersive Spectroscopy (EDS)

**Figure 6:**
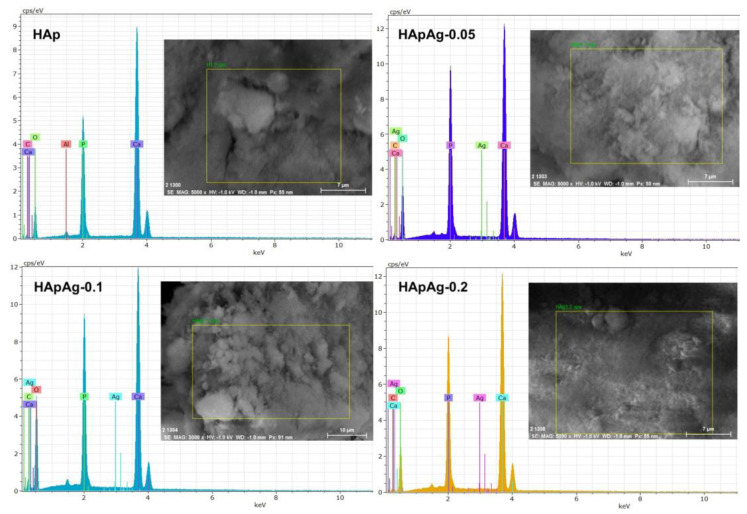
EDS spectra of the HApAg samples with xAg = 0, xAg = 0.05, xAg = 0.1, and xAg = 0.2.

EDS results are shown in Figure 6; the presence of peaks corresponding to the elements present, such as calcium (Ca), phosphorus (P), oxygen (O), and silver (Ag), can be observed. The presence of silver in the three doped samples suggests the incorporation of silver ions into the structure. In addition, the (Ca + Ag)/P ratio was determined in all the samples, showing a stoichiometric ratio of 1.82, which agrees with previous research [41]. Recent research shows that silver-doped hydroxyapatite obtained via hydrothermal synthesis presents a change in the C axis of the structure. This suggests an improvement in the physicochemical properties of the material as well as possible antibacterial applications in bone tissue regeneration [42,43]. All elements present show a homogeneous distribution in all samples examined via X-ray elemental analysis. EDS mapping confirmed that silver was randomly distributed throughout the sample. With the data obtained, it can be concluded that the silver-doped hydroxyapatite powders were highly homogeneous from the morphological and chemical points of view.

### 3.5. X-ray Diffraction (XRD)

X-ray diffraction was used to analyze the structure of the powders. Diffraction angle and intensity helped identify the structure. In addition, structural parameters of the material could be obtained, such as crystalline planes and crystallite size.

**Figure 7:**
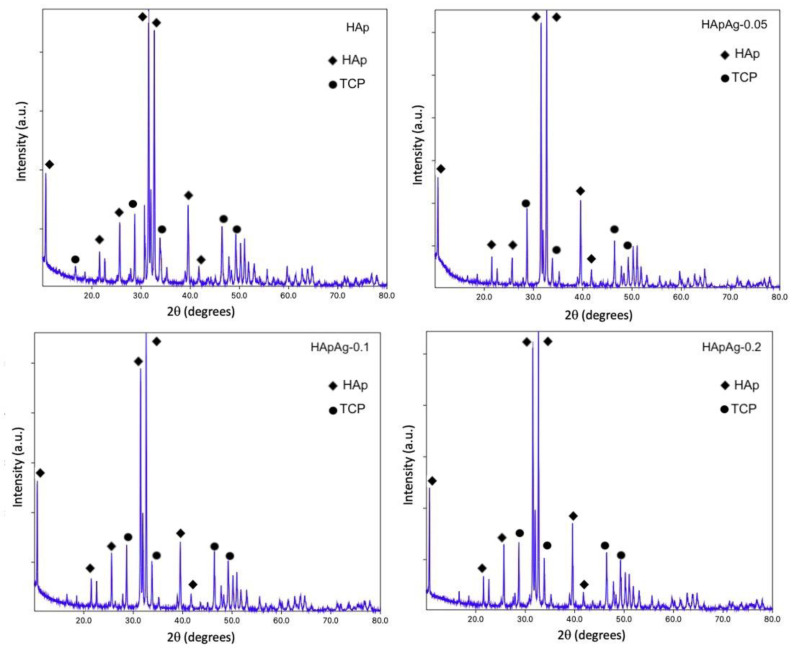
XRD patterns of HApAg samples with Ag = 0, Ag = 0.05, Ag = 0.1. and Ag = 0.2.

XRD analyses for all HApAg powders were conducted. Figure 7 shows the diffraction patterns for HAp, HApAg-0.05, HApAg-0.1, and HApAg-0.2. The results showed that the HApAg powders are totally crystalline, showing hydroxyapatite as the only phase present. It was observed that the phase present in the diffraction diagrams obtained for the HApAg powders coincides with the HAp diffraction pattern compared to the powder diffraction file (PDF) # 01-084-1998. The observed reflections correspond to the following families of planes for Hap” (100), (002), (210) (112), (300), (310), and (222) located at 10°, 20°, 25°, 32°, 33°, 40°, and 42° in 2θ, respectively. An intensity ratio index was established between all the peaks, resulting in a ratio of 0.67. The observed reflections for TCP correspond (102), (202), (312), and (213) located at 28°, 34°, 46°, and 50° in 2θ, respectively.

The crystallite size was calculated from the diffraction patterns using the Scherrer equation [44]. The results are shown in Table 5.

(1) $$D=\frac{K\cdot \lambda }{\beta \cdot cos\theta }$$

XRD studies of HApAg showed that the powders obtained using the sol-gel method present the characteristic structure of crystalline hydroxyapatite. For the HAp sample, an additional peak associated with the tricalcium phosphate (TCP) phase is observed because of the presence of carbonate. These results agree with FTIR and SEM, suggesting that the dopant has no influence on the crystalline structure and morphology of the powders.

In agreement with previous studies, the absence of another phase indicates that the doping agent (silver) replaced the calcium present in the structure without modifying the crystal lattice. On the other hand, the observed peaks are very sharp, and their intensity is proportional to the concentration of silver in the powders, which suggests a good crystallization of the hydroxyapatite [45].

### 3.6. Antibacterial Evaluation

**Figure 8:**
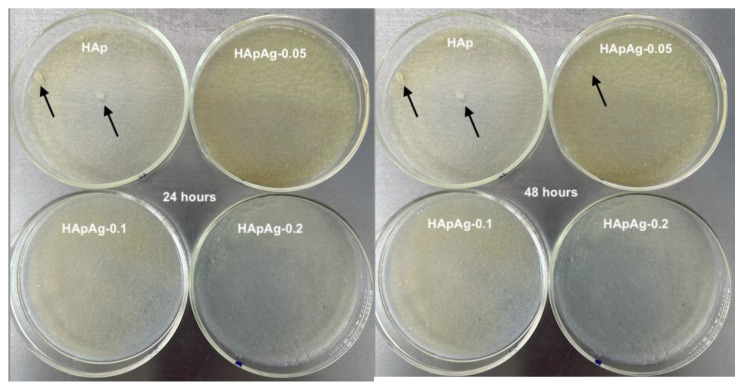
Culture media at 24 and 48 h of HApAg samples with Ag = 0, Ag = 0.05, Ag = 0.1, and Ag = 0.2. Black arrows indicate colony formation.

To evaluate possible differences in bacterial growth, an antibacterial evaluation was performed. Culture media with nutrient agar/*Escherichia coli* and HApAg powders were prepared and incubated. Figure 8 shows images of the culture media incubated at 24 and 48 h.

The results showed the growth of white bacteria after 24 h of incubation in the HAp sample, as shown in Figure 8, indicated by the arrows. At 48 h, a small growth of bacteria can also be observed in the HApAg-0.05 sample. The results obtained suggest that as the dopant (Ag) concentration increases, microbial growth is lower, as observed in the HApAg-0.1 and HApAg-0.2 samples that do not show bacterial growth. The results indicate that silver provides properties to prevent the growth of bacteria. Recent work showed that doped hydroxyapatite in low concentrations allows the antibacterial effect, but in high concentrations, it can cause cytotoxicity (more than 1.6 ppm) [46]. For the quantification of the bacteria present, the drip plate sealing methodology was used, obtaining the following values: 10^4^, 10^2^, 10^1^, and 10^1^ CFU/mL for HAp, HApAg-0.05, HApAg-0.1, and HApAg-0.2, respectively. Recent investigations have shown that the nanometric size of hydroxyapatite powders is a determining factor for the interaction with microbial cells, which produce a better antimicrobial effect [47,48]. Other authors have studied the inhibition of bacterial growth by nanoparticles with different morphology, showing that the antimicrobial efficiency depends on the shape of the nanostructures [48]. Recent studies have shown that the morphology of nanostructures plays an important role in the influence of silver to inhibit bacterial growth. Spherical nanostructures need 12 μg to inhibit bacterial growth, while rod-shaped nanostructures need 100 μg [48]. Our study showed that the antibacterial activity depends on the silver concentration, and the inhibitory effect increases proportionally as the silver concentration increases. However, more studies are needed to evaluate the cytotoxicity of silver-doped hydroxyapatite powders before suggesting in vivo applications.

## 4. Conclusions

A simple and low-cost methodology for obtaining silver-doped hydroxyapatite powders is described in this paper. Silver-doped hydroxyapatite was successfully synthesized using a precipitation method; the results were confirmed by FTIR, Raman, SEM, and EDS spectroscopy. In addition, in the analysis, a crystalline phase corresponding to hexagonal hydroxyapatite was identified in all the samples in addition to some peaks corresponding to the tricalcium phosphate phase, which indicates that the silver was well substituted in the lattice without modifying the crystalline structure. It was observed that the concentration of 0.2 M silver to perform the substitution is the ideal limit to not modify the structure. The physicochemical properties of the obtained powders suggest possible biomedical applications of the material. Furthermore, the antimicrobial evaluation suggests that silver-doped hydroxyapatite powders inhibit the growth of bacteria, having potential applications in bone tissue regeneration. The findings of the study represent an advance in the field of bone tissue regeneration; however, there are some limitations, such as the size of the sample evaluated and a possible long-term antimicrobial evaluation. Our work suggests future research to examine the long-term effects of silver-doped hydroxyapatite using different synthesis methods as well as biocompatibility tests. Likewise, it is necessary to carry out a Rietveld refinement analysis to identify the sites where silver ions are found within the structure. No human or animal tests were performed in this study; however, it is necessary to evaluate the ethical implications of the use of this material in living organisms.

## Figures and Tables

**Table 1:** Comparison of different methods to obtain silver-doped hydroxyapatite [27,28,29].

Method	Advantages	Disadvantages
Chemical method	Morphology control, low cost	Low reproducibility, phase mixing
Sol-gel	Particle homogeneity, energy saving	Cost of reagents, processing times
Mechanochemical method	No use of solvents, low energy consumption	Sensitive efficiency, energy expenditure in grinding
PLD method	Multilayer growth, homogeneous coatings	Sensitivity in doping, high costs

**Table 2:** Mass of the precursors used in the synthesis of HApAg.

Concentration Ag (mol)	Ca(NO_3_)_2_ (g)	NH_4_H_2_PO_4_ (g)	AgNO_3_ (g)
0	11.75	3.43	0
0.05	11.65	3.42	0.042
0.1	11.55	3.41	0.084
0.2	11.36	3.38	0.167

**Table 3:** Absorption bands observed and assignments for silver-doped hydroxyapatite powders [Reprinted/adapted with permission from 34].

Peak (cm^−1^)	Assignment
3590, 3490, 3100	Water stretching [*v_s_* (O-H)]
1650	Bending of water δ (O-H)
1200	Hydroxyl group HPO
1100	Stretching (*v*_3_ P-O)
870	Bending (*v*_1_ C-O)
520	Bending (*v*_4_ P-O)

**Table 4:** Raman shifts observed for silver-doped hydroxyapatite powders.

Peak (cm^−1^)	Raman Shifts
430, 467	Mode ($\nu_{2}$) of the ${PO}_{4}$ group
610	Mode ($\nu_{4}$) of the ${PO}_{4}$ group
980, 1013	Stretching mode ($\nu_{3}$) of the ${PO}_{4}$ group

**Table 5:** Crystallite size of silver-doped HAp.

Sample	Wavelength (Å)	Peak Width (°)	Crystallite Size (nm)	Lattice Constant a, b, c (Å)
HAp	1.54	0.19	50.12	9.14, 9.13, 6.72
HApAg-0.05	1.54	0.21	48.34	9.22, 9.22, 6.78
HApAg-0.1	1.54	0.20	49.53	9.21, 9.21, 6.79
HApAg-0.2	1.54	0.22	47.51	9.25, 9.25, 6.77

## Data Availability

The data presented in this study are available on request from the corresponding author.
